# The usefulness of pretreatment controlling nutritional status score for predicting recurrence in patients with esophageal squamous cell carcinoma undergoing neoadjuvant immunochemotherapy: A real-world study

**DOI:** 10.3389/fimmu.2022.1015365

**Published:** 2022-11-24

**Authors:** Jifeng Feng, Liang Wang, Xun Yang, Qixun Chen, Xiangdong Cheng

**Affiliations:** ^1^ Department of Thoracic Oncological Surgery, Zhejiang Cancer Hospital, Institute of Basic Medicine and Cancer (IBMC), Chinese Academy of Science, Hangzhou, China; ^2^ Zhejiang Provincial Research Center for Upper Gastrointestinal Tract Cancer, Zhejiang Cancer Hospital, Institute of Basic Medicine and Cancer (IBMC), Chinese Academy of Science, Hangzhou, China; ^3^ The Second Clinical Medical College, Zhejiang Chinese Medical University, Hangzhou, Zhejiang, China

**Keywords:** controlling nutritional status, esophageal squamous cell carcinoma, pathologic complete response, recurrence, neoadjuvant immunochemotherapy, disease-free survival

## Abstract

**Background:**

The controlling nutritional status (CONUT) score, as an immune-nutritional index, has been reported to be related to prognosis in several cancers. Neoadjuvant immunochemotherapy (nICT) is an emerging pattern for cancer treatment in recent years. However, the usefulness of CONUT in esophageal squamous cell carcinoma (ESCC) with nICT has not been reported so far. This study attempted to clarify the usefulness of CONUT in predicting disease-free survival (DFS) in ESCC with nICT.

**Methods:**

Two hundred sixteen ESCC patients receiving nICT between 2019 and 2021 were retrospectively enrolled. Based on CONUT, the patients were divided into two groups: low groups (score ≤ 2) and high (score ≥ 3) groups. The relationships between CONUT and clinical characteristics were estimated. Cox regression analyses with hazard ratios (HRs) and 95% confidence intervals (CIs) were also performed to evaluate the prognostic factors of DFS.

**Results:**

Fifty-nine (27.3%) patients achieved pathologic complete response (pCR), and 30 (13.9%) cases had a recurrence. There were 150 cases (69.4%) in low CONUT group and 66 cases (30.6%) in high CONUT group, respectively. The results revealed that vessel invasion (*P* = 0.037), postoperative pneumonia (*P* = 0.001), advanced ypT stage (*P* = 0.011), cTNM stage (*P* = 0.007), and ypTNM stage (*P* < 0.001) were significantly related to patients with a high CONUT score. A high pCR rate was found in patients with a low CONUT score (33.3% vs. 13.6%, *P* = 0.003), and a high recurrence rate was found in patients with a high CONUT score (24.2% vs. 9.3%, *P* = 0.004), respectively. Patients with a low CONUT score had a better 1-year DFS than those with a high CONUT score (90.7% vs. 75.8%, *P* = 0.004). Multivariate analyses indicated that the pretreatment CONUT score was an independent predictor regarding DFS (HR = 2.221, 95% CI: 1.067–4.625, *P* = 0.033).

**Conclusion:**

A better response and a lower recurrence were found in ESCC patients with a lower pretreatment CONUT. As a useful index for immune-nutritional status, the CONUT might be a reliable prognostic indicator in ESCC patients with nICT.

## Introduction

Esophageal cancer (EC) is one of the life-threatening diseases worldwide, which ranks the 10th and sixth, respectively, in terms of incidence and mortality (1, 2). More than 50% of EC occurs in China, which ranks the sixth and fourth, respectively, in morbidity and mortality (3). There are two main pathological types of ECs, and more than 90% of them in China are esophageal squamous cell carcinoma (ESCC) (3). Patients with ESCC are often diagnosed at locally advanced stages at the time of presentation. Although the therapeutic methods for ESCC have been improved in recent years, the treatment effect is still unsatisfactory (4). Therefore, there is a need to explore more and more reliable indicators with pretreatment variables to predict prognosis before treatment.

For those patients with locally advanced ESCC, the NCCN guidelines recommend neoadjuvant chemoradiotherapy (nCRT) as the golden standard treatment in recent years (5). Due to the differences of races and pathological types between the West and the East, neoadjuvant chemotherapy (nCT) is usually recommended in China and Japan (6). However, the treatment effect and prognosis of neoadjuvant therapy for patients with ESCC are still unsatisfactory. Recently, immunotherapy has become one of the important regimens and has achieved several remarkable results in patients with advanced EC (7, 8). Compared with chemotherapy, immunotherapy has achieved a better long-term survival based on the ATTRACTION-3 and KEYNOTE-181 studies. According to the CheckMate 577 study, moreover, adjuvant nivolumab is also recommended after nCRT in patients with EC (9). Following these encouraging results in advanced EC, several clinical studies were also conducted, and the results revealed that neoadjuvant immunochemotherapy (nICT) followed by surgery is safe and feasible (10–13).

Recently, more and more researchers focus on the relations between the immune-nutritional status and cancer (14). The controlling nutritional status (CONUT), as an immune-nutritional index deriving from peripheral blood variables of albumin (ALB), lymphocyte (LYM), and total cholesterol (TC), has been reported to be related to prognosis in various cancers, including ESCC (15–17). Moreover, pretreatment CONUT was also widely used as a prognostic index in other digestive tract cancers, such as gastric cancer (GC) and colorectal cancer (CRC) (18–20). These results clearly demonstrated that a poor prognosis in various cancers was related to a high CONUT. Moreover, a recent study analyzed the prognostic value of CONUT in advanced EC patients who were treated with immunotherapy (21). The results revealed that a high CONUT score was associated with a significantly worse prognosis in advanced EC. In addition, it has also been shown that CONUT correlates with neoadjuvant response to treatment in several cancers, such as GC and EC (22, 23).

To date, most published studies focus on the efficacy and safety of nICT in local advanced ESCC. No studies on cancer recurrence in ESCC after nICT have been reported. Moreover, prior to nICT treatment, there are no reliable and affordable indexes to predict recurrence in ESCC. Therefore, we herein aimed to verify the recurrence pattern after nICT and explore the predictive value of CONUT in predicting DFS in ESCC with nICT.

## Patients and methods

### Patient selection

From 2019 to 2021, patients with local advanced ESCC with nICT in our department were enrolled. The inclusion criteria were as follows: (1) aged 18–75 years, (2) ESCC confirmed by histology, (3) ECOG-PS 0-1, (4) clinical TNM stage II-IVA, (5) radical R0 resection after nICT, and (6) complete clinical data and follow-up records over 6 months. The exclusion criteria were as follows: (1) non-ESCC, (2) R1 or R2 resection after nICT, (3) accompanied by other infection, hematologic, or autoimmune disease, (4) associated with other previous or synchronous cancers, or (5) combined with other anticancer treatment. All patients signed the written informed consent. The study was performed in accordance with the Helsinki Declaration. This study was reviewed and approved by the ethics committee of Zhejiang Cancer Hospital (IRB-2020-320).

### Treatment protocols

The preoperative nICT treatment protocols were the same as in our previously published studies (13, 24). The patients were also notified of alternative treatment options (nCRT or nCT) when they signed the informed consent. All patients received two cycles of nICT every 3 weeks. The immunotherapy regimen was administered on day 1 with the following protocol: pembrolizumab—2 mg/kg, nivolumab—3 mg/kg, or sintilimab/tislelizumab/camrelizumab—200 mg. The chemotherapy regimen was albumin paclitaxel (days 1 and 8: 100 mg/m^2^) combined with carboplatin (day 1: area under the curve, AUC = 5 mg/ml/min). Surgical resection was usually performed 4–6 weeks after the last cycle of nICT. The McKeown or Ivor Lewis minimally invasive esophagectomy (MIE) with two‐ or three-field lymphadenectomy was the main surgical treatment in the current study (25). To date, the adjuvant treatment after nICT followed by surgery in patients with EC remains unclear. Adjuvant treatment in our institute included adjuvant immunotherapy and adjuvant radiotherapy with or without chemotherapy. According to published studies, adjuvant immunotherapy was recommended in EC patients after nCRT based on the CheckMate 577 study and the expert consensus in China (9, 26). Moreover, adjuvant radiotherapy with or without chemotherapy was also recommended in patients with advanced ypT stage (T3–T4) and/or ypN stage (N1-3) after radical resection (27, 28).

### Follow-up

All patients were followed up periodically after completion of the treatments. The patients were followed up every 3 months during the first 2 years. Patients with no recurrence during the next 3–5 years were generally followed up every 6 months and once a year thereafter. The 8th AJCC/UICC staging system was used in this study (29). Pathological complete response (pCR) was defined as no evidence of residual tumor cells (30). Recurrence was regarded as any local, regional, or distant tumor recurrence. Disease-free survival (DFS) was defined as the time from surgery to the first documented recurrence. The last follow-up time was completed in June 2022.

### CONUT definition

**Figure 1:**
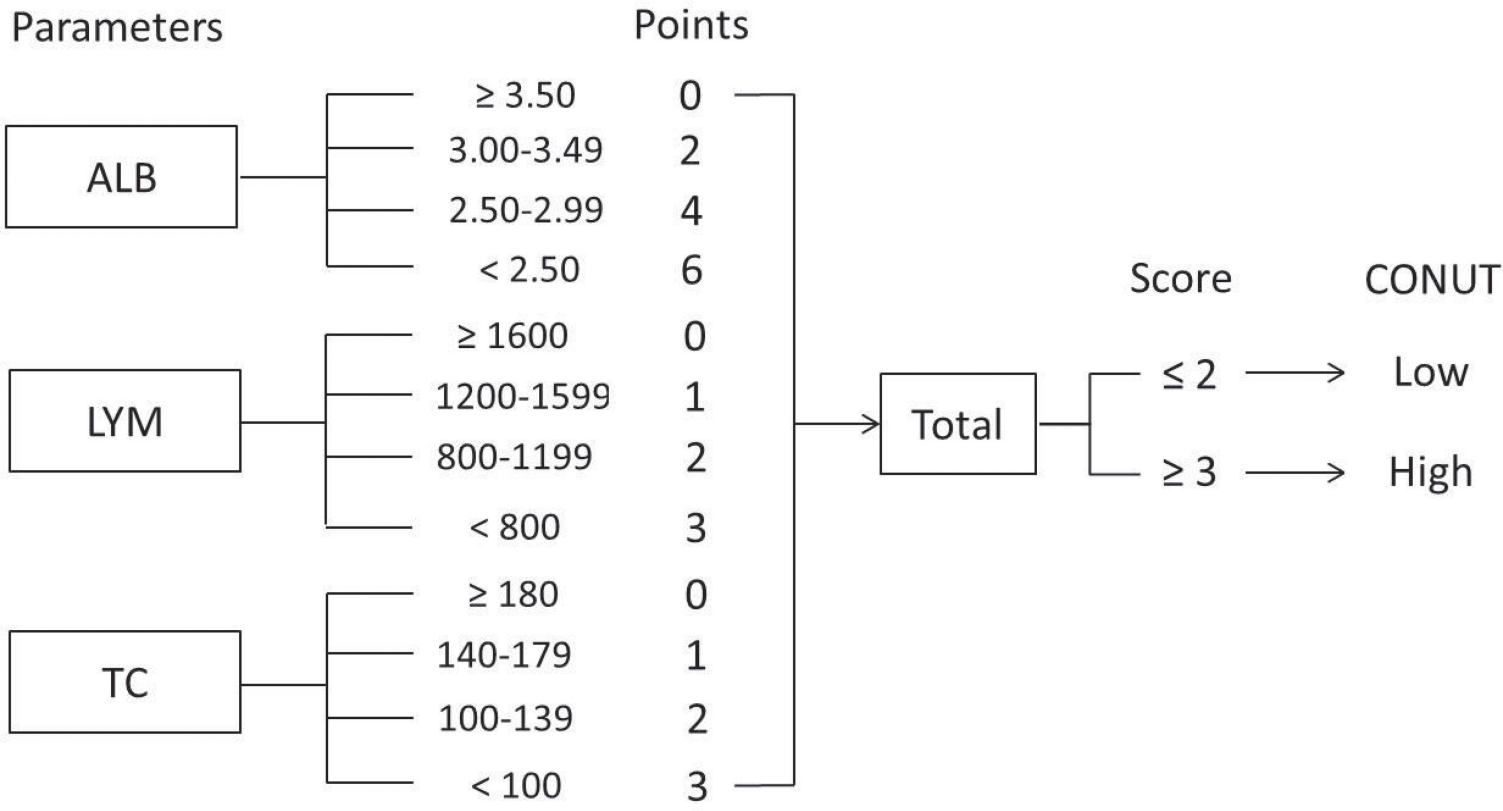
Flowchart for CONUT construction and risk stratification. The CONUT is calculated as the sum of the albumin, lymphocyte, and total cholesterol.

The CONUT score, according to various published studies, was based on three hematological indicators, including LYM, TC, and ALB (15–20). Based on previously published studies, the CONUT was usually collected within 1 week before treatment (15, 16, 18–20). Therefore, data on the levels of the abovementioned three hematological variables within 1 week before nICT were extracted. Then, the patients were divided into two groups based on CONUT score: low group (score ≤2) and high group (score ≥3). The flowchart for CONUT construction is shown in **Figure 1**.

### Statistical analysis

SPSS 20.0 and R software 4.1.2 were used to perform all statistical analyses. Student’s *t*-tests and chi-square or Fisher’s exact tests were carried out to analyze continuous and categorical variables, respectively. A three-dimensional scatter plot and a heat map were also drawn to explore the correlations. To better understand the predictive ability for pCR and recurrence, the AUCs were compared by receiver operating characteristic (ROC) curves. In the current study, clinical variables with statistical differences in the univariate analyses were then subjected to multivariate analyses by using a forward stepwise regression. Covariance analyses were also performed to avoid an interaction effect before Cox regression. Cox regression analyses with hazard ratios (HRs) and 95% confidence intervals (CIs) were used to identify the predictors of DFS. The DFS and overall survival (OS) differences were compared by log-rank tests in Kaplan–Meier curves. Finally, a novel nomogram model was also established to verify the prognostic value of independent prognostic factors. All statistical tests were two-sided, and a *p*-value <0.05 was considered to be statistically significant.

## Results

### Patient characteristics

A total of 216 ESCC patients with nICT were enrolled. The mean age of all patients was 63.2 ± 6.6 years (range: 47–75 years). There were 13 (6.0%) female and 203 (94.0%) male patients. The median time of follow-up was 12 months (range: 6–29 months). The majority of the ESCC was located in the middle (57.9%) and lower (32.9%) segment of the esophagus. Most types of differentiation were moderate (46.7%) and poor (37.5%). The ypTNM stages were as follows: 59 had stage 0 (27.3%), 63 had stage I–II (29.2%), and 94 had stage III–IVa (43.5%). There were 59 (27.3%) patients who achieved pCR, and 30 (13.9%) cases had a recurrence. The detailed clinical characteristics are shown in **Table 1**.

**Table 1:** Clinical characteristics in 216 esophageal squamous cell carcinoma patients.

Characteristics	Value
Age [mean ± SD (range), years]	63.2 ± 6.6 (47–75)
Sex (male/female, %)	203 (94.0)/13 (6.0)
ECOG-PS (0/1, %)	188 (87.0)/28 (13.0)
BMI [mean ± SD (range), kg/m^2^]	21.6 ± 2.3 (17.3–30.1)
Tumor length [mean ± SD (range), cm]	1.86 ± 2.02 (0–9.0)
Tumor location (upper/middle/lower, %)	20 (9.2)/125 (57.9)/71 (32.9)
Differentiation (well/moderate/poor, %)	32 (14.8)/101 (46.7)/81 (37.5)
Hypertension history (yes/no, %)	63 (29.2)/153 (70.8)
Diabetes history (yes/no, %)	8 (3.7)/208 (96.3)
Smoking history (yes/no, %)	153 (70.8)/63 (29.2)
Drinking history (yes/no, %)	158 (73.1)/58 (26.9)
Vessel invasion (yes/no, %)	22 (10.2)/194 (89.8)
Perineural invasion (yes/no, %)	37 (17.1)/179 (82.9)
Surgery type (McKewon/Ivor Lewis, %)	184 (85.2)/32 (14.8)
Operation time [mean ± SD (range), min]	217.6 ± 21.6 (175–310)
Stay after operation [mean ± SD (range), day]	13.3 ± 6.5 (9–52)
Blood loss [mean ± SD (range), ml]	149.5 ± 66.3 (50–400)
Anastomotic leak (yes/no, %)	20 (9.3)/196 (90.7)
Pneumonia (yes/no, %)	48 (22.2)/168 (77.8)
cTNM stage (T2/T3/T4a, %)	52 (24.1)/132 (61.1)/32 (14.8)
ypT stage (T0/T1-2/T3-4a, %)	63 (29.2)/75 (34.7)/78 (36.1)
ypN stage (N0/N1/N2-3, %)	131 (60.6)/53 (24.6)/32 (14.8)
ypTNM stage (0/I–II/III-Iva, %)	59 (27.3)/63 (29.2)/94 (43.5)
LYM [mean ± SD (range),/mm^3^]	1,552 ± 565 (500–5,100)
ALB [mean ± SD (range), g/dl]	4.06 ± 0.40 (2.75–4.98)
TC [mean ± SD (range), mg/dl]	172.9 ± 40.0 (97.8–270.7)
CONUT [mean ± SD (range)]	1.89 ± 1.60 (0–7)

ESCC, esophageal squamous cell carcinoma; SD, standard deviation; ECOG-PS, Eastern Cooperative Oncology Group Performance Status; BMI, body mass index; TNM, tumor node metastasis; LYM, lymphocyte; ALB, albumin; TC, total cholesterol; CONUT, controlling nutritional status.

### Correlations between CONUT scoreand components

**Figure 2:**
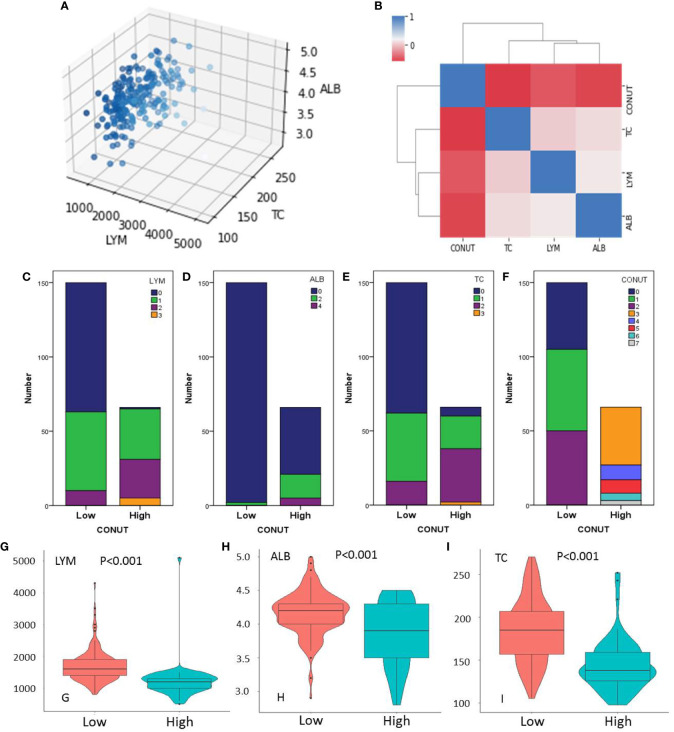
Three-dimensional scatter **(A)** and heat map diagram (**B**). Number of cases based on the lymphocyte (LYM) **(C)**, albumin (ALB) **(D)**, total cholesterol (TC) **(E)**, and CONUT **(F)**. The levels of LYM **(G)**, ALB **(H)**, and TC **(I)** grouped by CONUT.

The three-dimensional scatter diagram regarding the three variables is shown in **Figure 2A**. The mean values for LYM, ALB, and TC were 1,552 ± 565/mm^3^, 4.06 ± 0.40 mg/dl, and 172.9 ± 40.0 mg/dl, respectively. The heat map correlation diagram of CONUT and its components is shown in **Figure 2B**. According to our results, negative correlations were found between CONUT and LYM (*r* = -0.456, *P* < 0.001), ALB (*r* = -0.532, *P* < 0.001), and TC (*r* = -0.582, *P* < 0.001), respectively. A positive correlation was found between LYM and ALB (*r* = 0.164, *P* = 0.016). The number of cases based on the components of CONUT is shown in **Figures 2C–F**. The levels of LYM, ALB, and TC were significantly lower in the high CONUT group than those in the low CONUT group, respectively (*P* < 0.001, **Figures 2G–I**).

### AUC comparisons between CONUT and components

**Figure 3:**
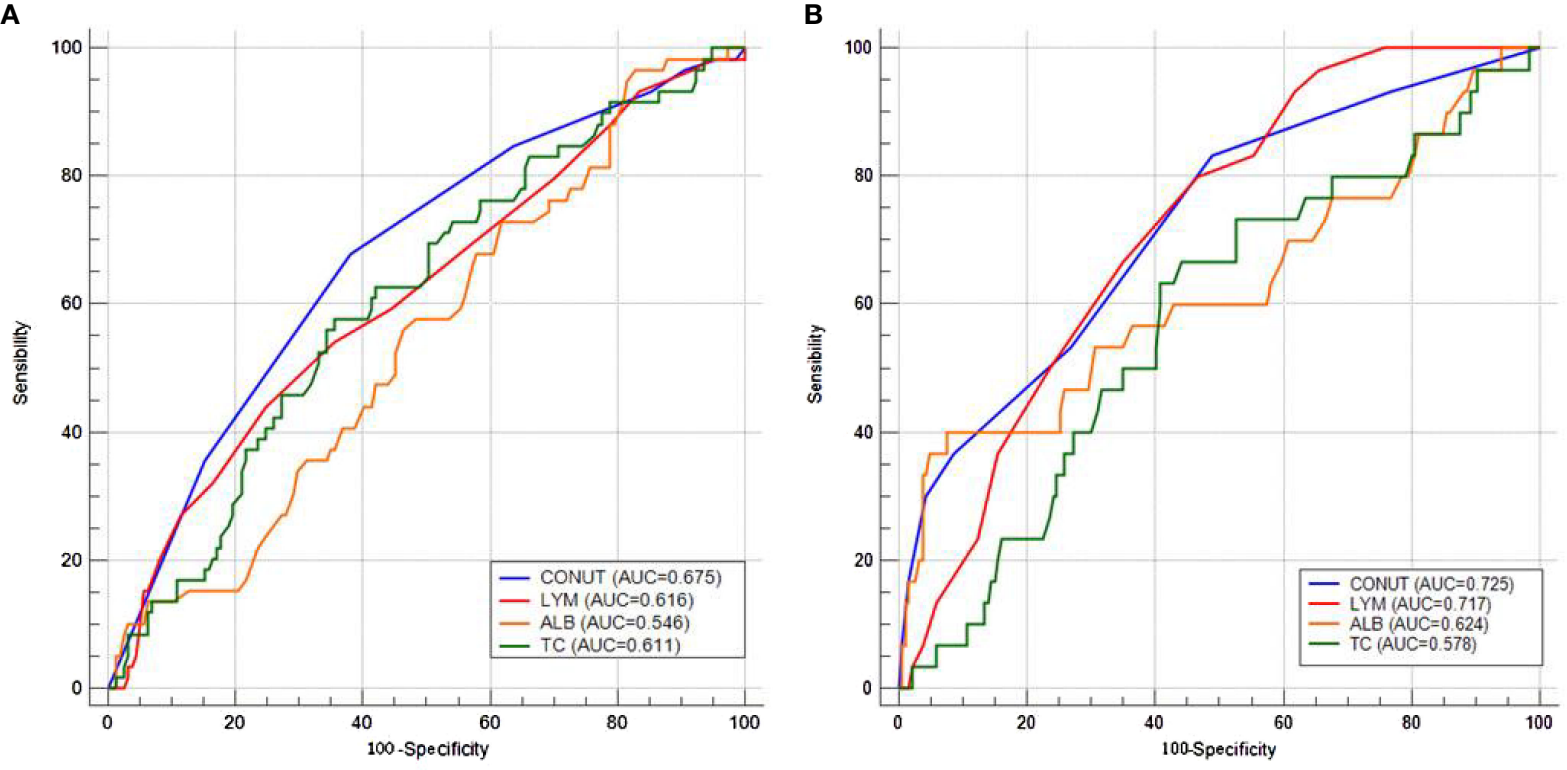
Area under the curve comparisons between CONUT and components (lymphocyte, albumin, and total cholesterol) according to the receiver operating characteristic curves in pathologic complete response **(A)** and recurrence **(B)** prediction.

The AUC comparisons between CONUT and components (LYM, ALB, and TC) according to the ROC curves in pCR and recurrence prediction are shown in **Figure 3**. Compared with its components of LYM, ALB, and TC, CONUT had the largest AUC (0.675 in pCR prediction and 0.725 in recurrence prediction) based on ROC curve analyses. As a comprehensive indicator, the CONUT can reflect host immune and nutritional status in a more extensive manner than other indicators. These results indicated a higher predictive ability of CONUT on pCR and recurrence prediction than other indicators.

### Patient characteristics grouped by CONUT

**Figure 4:**
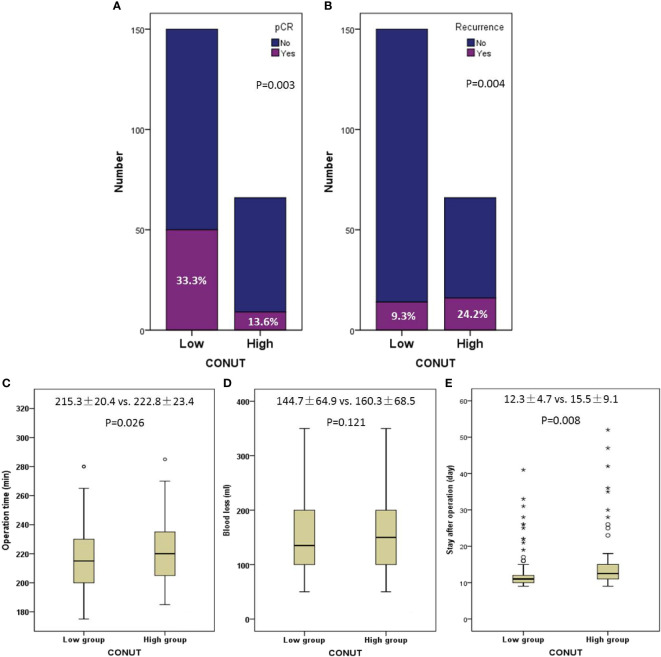
Comparisons of pathologic complete response (33.1% *vs*. 13.8%, *P* = 0.004) **(A)** and recurrence (24.6% vs. 9.3%, *P* = 0.003) **(B)** grouped by CONUT. Correlations between CONUT and operation time (*P* = 0.026) **(C)**, blood loss (*P* = 0.141) **(D)**, and hospital stay after operation (*P* = 0.008) **(E)**. The symbols of “*, ^O^” mean abnormal value.

One hundred fifty (69.4%) cases were enrolled in the low CONUT and 66 (30.6%) cases were enrolled in the high CONUT, respectively. Comparisons of the clinical characteristics grouped by CONUT are shown in **Table 2**. High CONUT was associated with vessel invasion (*P* = 0.037), postoperative pneumonia (*P* = 0.001), advanced ypT stage (*P* = 0.011), cTNM stage (*P* = 0.007), and ypTNM stage (*P* < 0.001). Moreover, a high pCR rate was found in the low CONUT group (33.3% vs. 13.6%, *P* = 0.003), and a high recurrence rate was found in the high CONUT group (24.2% vs. 9.3%, *P* = 0.004), respectively (**Figures 4A, B**). In addition, the pretreatment CONUT score was also significantly associated with operation time (*P* = 0.026) and hospital stay after the operation (*P* = 0.008) but not connected with blood loss (**Figures 4C–E**).

**Table 2:** Comparison of the clinical variables in esophageal squamous cell carcinoma grouped by CONUT.

	Low CONUT (*N* = 150)	High CONUT (*N* = 66)	*P*-value
Age (≤70/>70, years)	123 (82.0)/27 (18.0)	53 (80.3)/13 (19.7)	0.767
Sex (female/male)	11 (7.3)/139 (92.7)	2 (3.0)/64 (97.0)	0.221
ECOG-PS (0/1)	132 (88.0)/18 (12.0)	56 (84.8)/10 (15.2)	0.525
BMI (≤20/>20, kg/m^2^)	39 (26.0)/111 (84.0)	17 (25.8)/49 (74.2)	0.97
Tumor length (≤3/>3, cm)	111 (74.0)/39 (26.0)	54 (81.8)/12 (18.2)	0.213
Tumor location (U/M/L)	13 (8.7)/86 (57.3)/51 (34.0)	7 (10.6)/39 (59.1)/20 (30.3)	0.818
Differentiation (W/M/P)	26 (17.3)/66 (44.0)/58 (38.7)	6 (9.1)/35 (53.0)/25 (37.9)	0.234
Hypertension history (no/yes)	105 (70.0)/45 (30.0)	48 (72.7)/18 (27.3)	0.685
Diabetes history (no/yes)	146 (97.3)/4 (2.7)	62 (93.9)/4 (6.1)	0.224
Smoking history (no/yes)	48 (32.0)/102 (68.0)	15 (22.7)/51 (77.3)	0.167
Drinking history (no/yes)	38 (17.9)/112 (82.1)	20 (30.3)/46 (69.7)	0.448
Vessel invasion (no/yes)	139 (92.7)/11 (7.3)	55 (83.3)/11 (16.7)	0.037
Perineural invasion (no/yes)	128 (85.3)/22 (14.7)	51 (77.3)/15 (22.7)	0.148
Surgery type (M/I)	129 (86.0)/21 (14.0)	55 (83.3)/11 (16.7)	0.611
Anastomotic leak (no/yes)	139 (92.7)/11 (7.3)	57 (86.4)/9 (13.6)	0.141
Pneumonia (no/yes)	126 (84.0)/24 (16.0)	42 (63.6)/24 (36.4)	0.001
cTNM stage (T2/T3/T4)	44 (29.3)/89 (59.3)/17 (11.4)	8 (12.1)/43 (65.2)/15 (22.7)	0.007
ypT stage (T0/T1-2/T3-4a)	53 (35.3)/47 (31.4)/50 (33.3)	10 (15.2)/28 (42.4)/28 (42.4)	0.011
ypN stage (N0/N1/N2-3)	91 (60.7)/39 (26.0)/20 (13.3)	40 (60.6)/14 (21.2)/12 (18.2)	0.561
ypTNM stage (0/I-II/III-Iva)	50 (33.3)/33 (22.0)/67 (44.7)	9 (13.6)/30 (45.5)/27 (40.9)	<0.001

ESCC, esophageal squamous cell carcinoma; CONUT, controlling nutritional status; ECOG-PS, Eastern Cooperative Oncology Group Performance Status; BMI, body mass index; TNM, tumor node metastasis; U/M/L, upper/middle/lower; W/M/P, well/moderate/poor; M/I, McKewon/Ivor Lewis.

### Patterns of recurrence site

**Figure 5:**
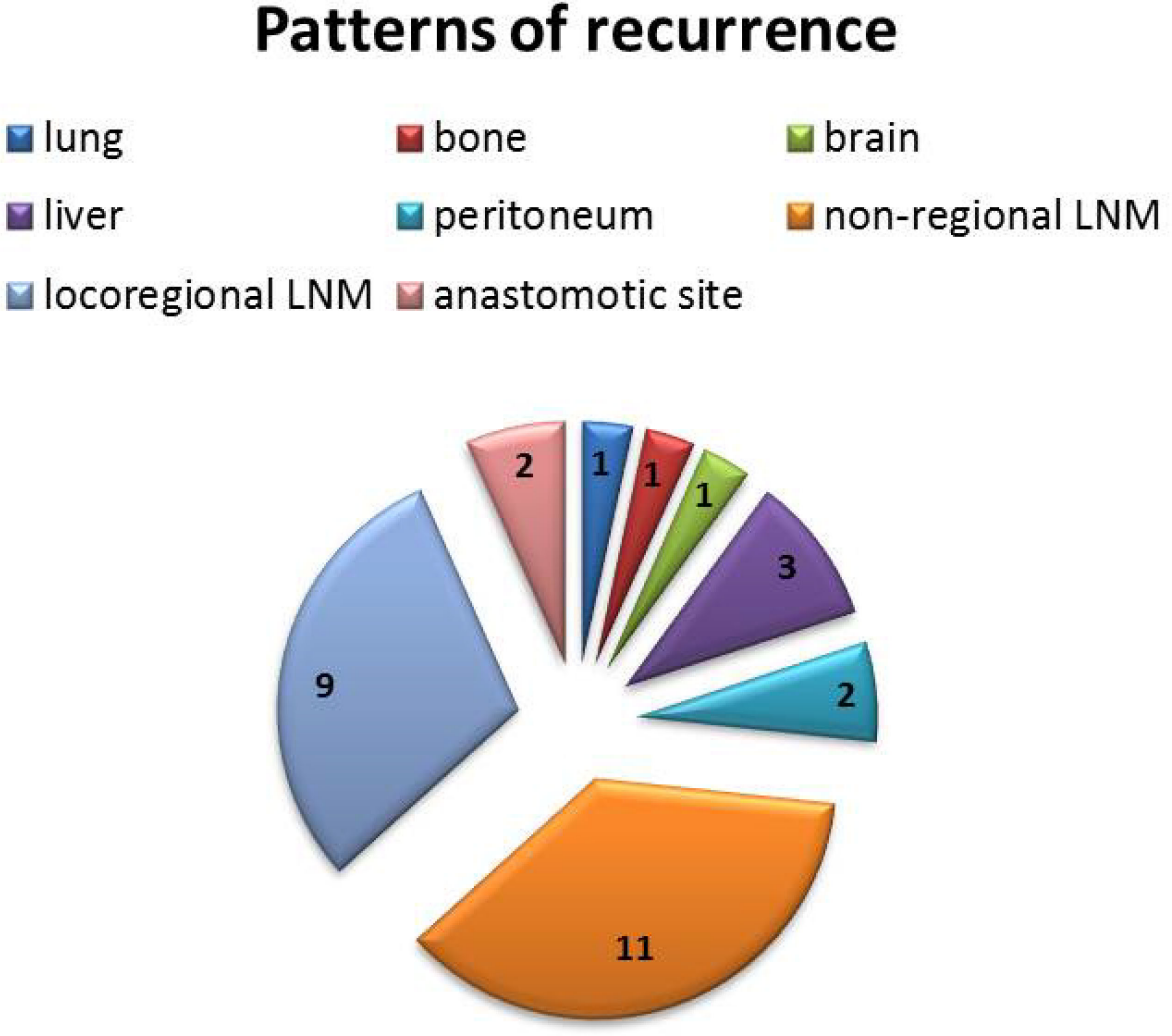
Detailed recurrence patterns after neoadjuvant immunochemotherapy. There were 19 (63.3%) cases with distant recurrence and 11 (36.7%) cases with local recurrence.

Depending on the initial presentation, the patients were divided into local recurrence and distant recurrence, respectively. There were 19 (63.3%) patients with distant recurrence after treatment, including peritoneal metastasis and non-regional lymph node metastasis (LNM), while there were 11 (36.7%) cases with local recurrence, including locoregional LNM and anastomotic site recurrence. The detailed recurrence patterns are shown in **Figure 5**. The recurrence was confirmed by biopsy at the anastomotic site in two patients, and the remaining recurrences were scored by using imaging examinations.

### Predictors to recurrence with Cox analyses

Clinical characteristics were used to perform Cox regression analyses (**Table 3**). There were nine variables, including location, tumor length, vessel invasion, perineural invasion, pCR, CONUT, ypT stage, ypN stage, and ypTNM stage, which were associated with DFS in the univariate analyses. The variables distinguished in the univariate analyses were then recruited in multivariate analyses. Multivariate Cox analyses indicated that tumor location (*P* = 0.006), ypN stage (*P* < 0.001), and CONUT (*P* = 0.033) were independent predictors regarding DFS. Patients in the high CONUT group had an HR of 2.221 (95% CI: 1.067–4.625) for DFS.

**Table 3:** Cox analyses of predictors for disease-free survival in esophageal squamous cell carcinoma.

	Univariate analysis		Multivariate analysis	
	HR (95% CI)	P-value	HR (95% CI)	P-value
Age (years, >70 vs. ≤70)	0.141 (0.019-1.036)	0.054		
Sex (male vs. female)	0.520 (0.157-1.717)	0.283		
ECOG-PS (1 vs. 0)	1.066 (0.372-3.055)	0.906		
BMI (Kg/m^2^, >20 vs. ≤20)	1.170 (0.502-2.728)	0.716		
Tumor length (cm, >3 vs. ≤3)	2.226 (1.055-4.694)	0.036		
Tumor location		0.013		0.006
upper	Reference		Reference	
middle	0.245 (0.096-0.623)	0.003	0.245 (0.095-0.632)	0.004
lower	0.403 (0.156-1.042)	0.061	0.231 (0.086-0.624)	0.004
Differentiation		0.235		
well	Reference			
moderate	5.161 (0.681-39.084)	0.112		
poor	5.815 (0.765-44.231)	0.089		
Hypertension history (yes vs. no)	0.605 (0.247-1.481)	0.272		
Diabetes history (yes vs. no)	0.804 (0.109-5.903)	0.830		
Smoking history (yes vs. no)	1.104 (0.491-2.482)	0.811		
Drinking history (yes vs. no)	1.511 (0.618-3.698)	0.366		
Vessel invasion (yes vs. no)	3.457 (1.475-8.104)	0.004		
Perineural invasion (yes vs. no)	2.298 (1.052-5.021)	0.037		
ypT stage		0.020		
T0	Reference			
T1-2	2.909 (0.801-10.572)	0.105		
T3-4a	5.259 (1.540-17.961)	0.008		
ypN stage		<0.001		<0.001
N0	Reference		Reference		
N1	3.104 (1.043-9.242)	0.042	3.309 (1.099-9.957)	0.033
N2-3	16.071 (6.305-40.968)	<0.001	16.838 (6.378-44.455)	<0.001
				
ypTNM stage		<0.001	
0	Reference			
I-II	0.930 (0.188-4.606)	0.929		
III-IVa	5.826 (1.753-19.364)	0.004		
pCR (yes vs. no) CONUT (high vs. low)	0.273 (0.083-0.900) 2.736 (1.335-5.607)	0.033 0.006	2.221 (1.067-4.625)	0.033
Radiotherapy (yes vs. no)	0.982 (0.376-2.567)	0.971		

ESCC, esophageal squamous cell carcinoma; DFS, disease-free survival; ECOG-PS, Eastern Cooperative Oncology Group Performance Status; BMI, body mass index; TNM, tumor node metastasis; CONUT, controlling nutritional status; pCR, pathological complete response; HR, hazard ratio; CI, confidence interval.

### DFS analyses and postoperative therapy

**Figure 6:**
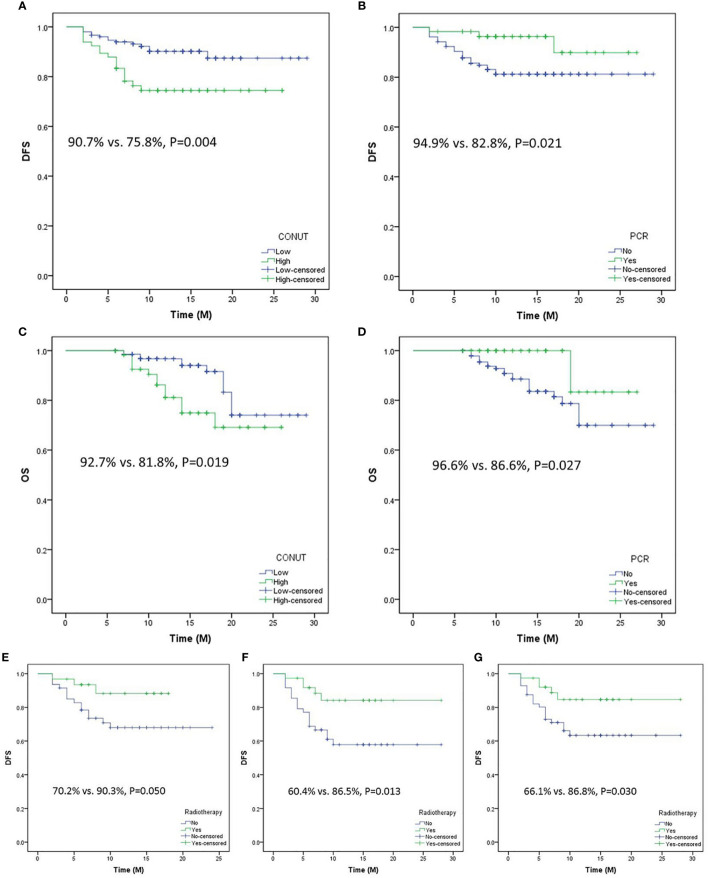
Disease-free survival (DFS) analyses grouped by CONUT **(A)** and pathologic complete response (pCR) **(B)**. Overall survival analyses grouped by CONUT **(C)** and pCR **(D)**. Subgroup analyses regarding DFS grouped by postoperative radiotherapy or not in ypT3-T4a **(E)**, yp N1-3 **(F)**, and ypTNM III-IVa **(G)**.

Patients with low CONUT had a better 1-year DFS than those with high CONUT (90.7% *vs*. 75.8%, *P* = 0.004, **Figure 6A**). Compared with patients with pCR, patients in the non-pCR group had a worse 1-year DFS (94.9% vs. 82.8%, *P* = 0.021, **Figure 6B**). Moreover, better OS curves were also found in patients with low CONUT (92.7% vs. 81.8%, *P* = 0.019, **Figure 6C**) and pCR (96.6% vs. 86.6%, *P* = 0.027, **Figure 6D**). No significant differences were found in this study between patients with or without postoperative radiotherapy. However, a subgroup analysis revealed that patients in the advanced stage with postoperative radiotherapy had a better 1-year DFS than those without radiotherapy (ypT3-4a: 90.3% vs. 70.2%, *P* = 0.050; ypN1-3: 86.5% vs. 60.4%, *P* = 0.013; ypTNM III-IVa: 86.8% vs. 66.1%, *P* = 0.030, respectively) (**Figures 6E–G**).

### Nomogram model established for recurrence prediction

**Figure 7:**
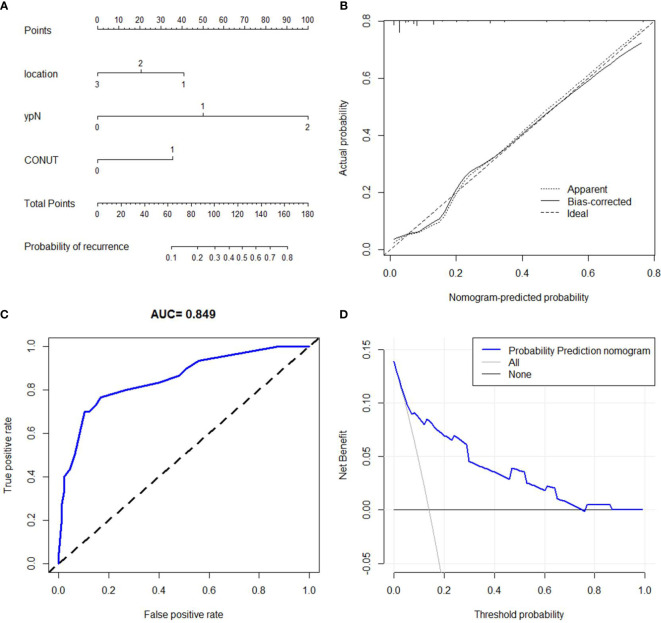
Nomogram for recurrence prediction. A predictive nomogram with the C-index of 0.846 was established **(A)**. The calibration revealed an acceptable agreement of recurrence prediction internally **(B)**. Receiver operating characteristic **(C)** and decision curve analyses **(D)** indicated a good clinical applicability of the model in predicting recurrence.

A predictive nomogram of recurrence prediction in ESCC with nICT was established based on location, ypN stage, and CONUT (**Figure 7A**). The C-index for the nomogram model was 0.846. The model was confirmed through 1,000 bootstrapping internal validation, indicating an optimal agreement between the actual observation and model prediction (C-index: 0.838, **Figure 7B**). A good predictive ability regarding recurrence was also found according to the ROC and decision curve analyses (**Figures 7C, D**).

## Discussion

In the current study, we initially explored the role of pretreatment CONUT on recurrence prediction in ESCC with nICT. The results revealed that the CONUT score may be used as a predictor for DFS and OS. In addition, a nomogram was also developed to predict recurrence after nICT. To our knowledge, this is the first report that focused on the role of CONUT score in predicting recurrence after nICT in ESCC. Moreover, we initially proposed a predictive model to predict recurrence after nICT for ESCC. The results of our study will have an important significance to local advanced ESCC patients who were treated with nICT.

In recent years, immunotherapy has become the focus of cancer treatment. Recent studies revealed that immunotherapy significantly improved the prognosis in several randomized phase III studies and was approved for first-line treatment for advanced ESCC (7, 8). As an exploratory attempt, nICT has already been investigated for ESCC in several studies. The results as well as our previously published study indicated that a high R0 resection and pCR rate were found in patients with ESCC receiving nICT (10–13). However, most previously published studies focused on the efficiency and safety of nICT. There are no studies regarding recurrence after nICT in patients with ESCC so far. Therefore, it is important to understand the real treatment effect of nICT in patients with ESCC.

As a useful index for immune-nutritional status, recent studies revealed that CONUT score was an effective predictive and prognostic indicator in various cancers (15–17). Since 2016, a few studies on the CONUT score in patients with EC have been reported (16, 23, 31–33). A study including 352 ESCC patients with surgery reported that patients with moderate or severe CONUT score were at a high risk of postoperative complications (31). Another study revealed that patients with moderate or severe CONUT score were related to poor prognosis in 373 ESCC patients with radical resection (16). Compared with other indicators, several studies have also shown the superiority of the CONUT score in predicting the prognosis in ESCC patients undergoing surgery (32, 33). In addition, some studies have evaluated the treatment response of pretreatment CONUT for neoadjuvant therapy in GC and EC patients (22, 23). Recently, a meta-analysis with 952 patients including five studies verified the significant associations between the CONUT and prognosis (34). However, no relevant research has yet been reported regarding the predictive value of the CONUT for response to nICT in ESCC.

A recent study analyzed the prognostic value of CONUT in advanced EC patients who were treated with immunotherapy (21). The results revealed that a high CONUT score was associated with a significantly worse prognosis. Similar results were also found in our study for patients with local advanced ESCC in nICT. Based on these results, the significance of CONUT score as a prognostic index is clearer. The results will bring an important assessment of recurrence pattern before nICT and help clinicians provide a more personalized approach to adjuvant therapy in ESCC after nICT. We believe that patients with high CONUT in ESCC should be regarded with caution. Adjuvant therapy may be required for those with high CONUT.

There are several potential mechanisms that could explain the relationship between CONUT and cancer prognosis. It is well known that CONUT comes from three hematological variables, representing caloric consumption, protein reservation, and impaired immune defense, respectively. LYM, as a determinant of immunity, can inhibit the proliferation, migration, and invasion of cancer cells by initiating a cytotoxic immune response (35, 36). Lymphocytopenia causes an insufficient host immune response, resulting in a poor prognosis in cancers (37). ALB is used as a nutritional score to reflect the nutritional status. It has been reported that the mechanism of hypoproteinemia leading to poor prognosis may be through the release of a variety of inflammatory cytokines, such as IL-6 and TNF-α (38). TC, as a vital component of the cell membrane, participates in various biological signaling pathways. The main effect of hypocholesterolemia on the ability of transmembrane signaling may be due to the increased uptake of TC by tumor cells (39). Thus, combined with these three components, CONUT may provide a better balance of immunological and nutritional status.

Some limitations should be recognized. Firstly, this was a single-center study with retrospective characteristics. Secondly, the CONUT score, as a hematological index, may be affected by various other conditions. Thirdly, although internal validation was verified, there was a lack of an external validation cohort to validate the nomogram. Fourthly, the follow-up time for this study was too short. Therefore, some bias may exist in the prognostic factors. Fifthly, there is no special stratified analysis in the current study due to the short follow-up time. Finally, the basic biological mechanisms with regard to CONUT have not been thoroughly elucidated. However, it is believed that, with more and more studies regarding nICT in ESCC, the CONUT will be better elucidated.

## Conclusion

These real-world data revealed that patients with a low score might have a better response and a lower recurrence. As a useful index for immune-nutritional status, the pretreatment CONUT score might be a reliable predictor in ESCC with nICT. The simple and easily obtained feature of the CONUT improves its application in daily clinical work.

## Data availability statement

The original contributions presented in the study are included in the article/**Supplementary Materials**. Further inquiries can be directed to the corresponding authors.

## Ethics statement

This study was performed in accordance with the Helsinki Declaration. This study was reviewed and approved by the ethics committee of Zhejiang Cancer Hospital (IRB-2020-320). The patients/participants provided their written informed consent to participate in this study.

## Author contributions

JF, QC, and XC conceived and designed the study. JF and LW collected the clinical baseline characteristics and drafted the manuscript. LW and XY carried out the follow-up. JF, LW, and XY performed the data analyses. QC and XC helped to draft the manuscript. All authors contributed to the article and approved the submitted version.

## Funding

This study was supported by Zhejiang TCM Science and Technology Project (2020ZB036, 2021ZB034, and 2022ZB051).

## Acknowledgments

The authors thank all the patients and their families who participated in this study.

## Conflict of interest

The authors declare that the research was conducted in the absence of any commercial or financial relationships that could be construed as a potential conflict of interest.

## Publisher’s note

All claims expressed in this article are solely those of the authors and do not necessarily represent those of their affiliated organizations, or those of the publisher, the editors and the reviewers. Any product that may be evaluated in this article, or claim that may be made by its manufacturer, is not guaranteed or endorsed by the publisher.
